# Bioinformatics Analysis Using ATAC-seq and RNA-seq for the Identification of 15 Gene Signatures Associated With the Prediction of Prognosis in Hepatocellular Carcinoma

**DOI:** 10.3389/fonc.2021.726551

**Published:** 2021-10-25

**Authors:** Hui Yang, Gang Li, Guangping Qiu

**Affiliations:** Department of Interventional Therapy, Hwa Mei Hospital, University of Chinese Academy of Science, Ningbo, China

**Keywords:** chromatin accessibility, ATAC-seq, hepatocellular liver cancer, LASSO model, prognosis

## Abstract

**Background:**

Gene expression (RNA-seq) and overall survival (OS) in TCGA were combined using chromosome accessibility (ATAC-seq) to search for key molecules affecting liver cancer prognosis.

**Methods:**

We used the assay for transposase-accessible chromatin with high-throughput sequencing (ATAC-seq) to analyse chromatin accessibility in the promoter regions of whole genes in liver hepatocellular carcinoma (LIHC) and then screened differentially expressed genes (DEGs) at the mRNA level by transcriptome sequencing technology (RNA-seq). We obtained genes significantly associated with overall survival (OS) by a one-way Cox analysis. The three were screened by taking intersection and further using a Kaplan–Meier (KM) for validation. A prognostic model was constructed using the obtained genes by LASSO regression analysis.The expression of these genes in hepatocellular carcinomas was then analysed. The protein expression of these genes was verified using the Human Protein Atlas(HPA) online datasets and immunohistochemistry.

**Results:**

ATAC-seq, RNA-seq and survival analysis, combined with a LASSO prediction model, identified signatures of 15 genes (*PRDX6, GCLM, HTATIP2, SEMA3F, UCK2, NOL10, KIF18A, RAP2A, BOD1, GDI2, ZIC2, GTF3C6 SLC1A5, ERI3* and *SAC3D1*), all of which were highly expressed in hepatocellular carcinoma. The LASSO prognostic model showed that this risk score had high predictive accuracy for the survival prognosis at 1, 3 and 5 years. A KM curve analysis showed that high expression of all 15 gene signatures was significantly associated with a poor prognosis in LIHC patients. HPA analysis of protein expression showed that *PRDX6*, *GCLM*, *HTATIP2*, *NOL10*, *KIF18A*, *RAP2A* and *GDI2* were highly expressed in the hepatocellular carcinoma tissues compared with normal control tissues.

**Conclusions:**

PRDX6, GCLM, HTATIP2, SEMA3F, UCK2, NOL10, KIF18A, RAP2A, BOD1, GDI2, ZIC2, GTF3C6, SLC1A5, ERI3 and SAC3D1 may affect the prognosis of LIHC.

## Introduction

According to Global Cancer Statistics 2020, hepatocellular liver cancer has the seventh-highest incidence rate but the second-highest mortality rate after lung cancer. As a common malignancy, with potentially fatal consequences, hepatocellular carcinomas have been widely studied (1). Although much research has focused on understanding hepatocellular cancer at the molecular level and therapeutic strategies have been developed, the biological mechanisms of hepatocarcinogenesis remain unclear. Due to slow progress in liver cancer research (2, 3), the patient survival rate remains low (i.e. < 8 months) (4, 5). Liver cancer is a more complex disease than other cancers, as its progression includes genetic modification processes, including gene mutations, gene deletions, translocations and DNA methylation (6). Therefore, early diagnosis and treatment are essential for improving the prognosis of liver cancer. To date, the only effective diagnostic method is detection of the serum tumour marker alpha-fetoprotein, which has an upper limit of normal value of 20 ng/mL (7). However, alpha-fetoprotein is nonspecific and has little statistical significance when detected in patients with different types of liver cancer (8). It is also ineffective for the diagnosis of early-stage liver cancer (9).

In eukaryotic cells, nuclear DNA and proteins combine to form chromatin, which then undergoes complex and orderly folding to form chromosomes (10). For genes to be expressed, chromatin must be in an open conformation. Open chromatin allows regulatory proteins to bind to DNA and regulate DNA function (11). The assay for transposase-accessible chromatin with high-throughput sequencing (ATAC-seq) enables high-throughput sequencing of open chromatin regions with the help of transposases. This simple method, which is very similar to ChIP-seq, requires only a small number of samples to obtain clear and reproducible sequencing results (12). ATAC-seq detects chromatin accessibility of related genes and indicates their regulatory mechanisms. Thus, genes with chromatin accessibility in promoters are more likely to be differentially expressed at the mRNA level and regulated by transcription factors (13).

Due to the complexity of gene expression regulatory mechanisms, it is crucial to be able to probe biological questions at different levels. Therefore, the integration and analysis of multi-omics is increasingly important. Differentially expressed genes (DEGs) can be analysed using RNA-seq and Chip-seq (14). Using Chip-seq, the regulatory role of specific transcription factors can be studied (15). ATAC-seq can shed light on the dynamics of chromatin accessibility. As chromatin accessibility is closely related to the binding of regulatory elements or transcription factors, it plays an important role in transcriptional regulation (12). Therefore, integration analysis can further explore the key factors of a biological process, as well as the target genes of a transcription factor. Currently, integration analysis studies combining ATAC-seq and RNA-seq are uncommon, with no such studies conducted on hepatocellular carcinomas. Therefore, in this study, we constructed a 15 gene signatures for predicting the prognosis in hepatocellular carcinoma patients by analysing and integrating ATAC-seq and RNA-seq.

## Methods

### Data Sources

ATAC-seq data on LIHC were obtained from the database of the University of California Santa Cruz (UCSC) (https://xenabrowser.net/datapages/). In total, ATAC-seq data of 404 LIHC samples were obtained, 17 of which were from TCGA database. The data were downloaded in promoter peak data format, with normalized correction. The calibration process included count conversion to CPM, after a base 2 logarithmic transformation.

RNA-seq data on LIHC were downloaded from the TCGA database, with 371 tumour samples and 50 para cancer samples.

### Chromatin Accessibility Analysis Using ATAC-seq

To explore the accessibility of chromatin, we first used the R package chromosome locator to show the peak regions on chromosomes. Peaks that could be mapped to TSS regions were aligned using the R-packaged ChIPseeker to construct a marker matrix. The nearest TSS region was selected for peak annotation. The annotation information was obtained from the R software. The relationship between open chromatin and promoter regions was revealed by UpSet plots.

### Analysis of DEGs Using RNA-seq

To assets differential expression of mRNA, the Limma package of R software (version: 3.40.2) was used. Adjusted *P* values in the TCGA dataset were analysed to correct for false-positive results. DEGs were obtained by screening with |log2(FC)| > 1 (*P* < 0.05). Heat and volcano plots were plotted using the R package ggplot2.

### Gene Oncology and Kyoto Encyclopedia of Genes and Genomes Enrichment Analysis

Peaks-associated genes were analysed by functional enrichment analysis. The ClusterProfiler package in R was used to analyse the GO/KEGG enrichment pathway of potential peaks-associated genes.

### Survival Analysis

The R package Survival was used for the survival analysis. The correlation between the expression levels of all known genes in LIHC and the overall survival (OS) of patients with hepatocellular liver cancer was analysed by a one-way Cox regression analysis, reporting hazard ratios (HRs) and their 95% confidence intervals (CIs). A Kaplan–Meier (KM) test was performed to analyse the difference between the survival of patients with high and low gene expression.

### LASSO Model

To compare survival differences between multiple groups, the log-rank test was used to test the KM survival analysis, and ROC analysis was used to compare gene prediction accuracy and risk scores. A LASSO regression was used for feature selection, with 10-fold cross-validation. The R package glmnet was used for the above analyses.

### Immune Cell Infiltration

The TIMER database (http://timer.comp-genomics.org/) was used to analyse the correlation of gene expression in LIHC with the level of immune cell infiltration.

### Protein Expression Validation

Immunohistochemical staining maps of gene signatures for protein expression in both liver cancer tissue and normal tissue were downloaded from The Human Protein Atlas (HPA) database.

## Results

### Identification of Chromatin Open Regions by ATAC-seq

**Figure 1:**
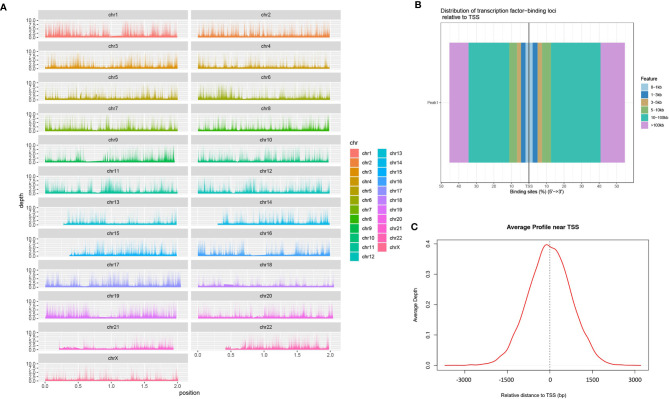
Identification of chromatin open regions by ATAC-seq method. [**(A)** genome coverage; **(B)** distribution map of transcription factors and TSS; **(C)** enrichment of Peaks in the TSS region].

We mapped the genomic coordinates from the Peak data to the 23 chromosomes of the human genome (**Figure 1A**). As can be seen, most regions of each chromosome are covered, with some chromosomes, such as chr13, chr14, chr21 and chr22, having less coverage of the short arms. **Figure 1B** shows that most of the peaks are concentrated at a distance of 10–100 kb from the TSS. Among these, the binding site tend to be distributed more at the 3’ end of the TSS. **Figure 1C** shows a large proportion of ATAC-peaks are located close to TSS, which means that the TSS tends to bind to transcription factors.

### Genomic Characterization and Enrichment Analysis

**Figure 2:**
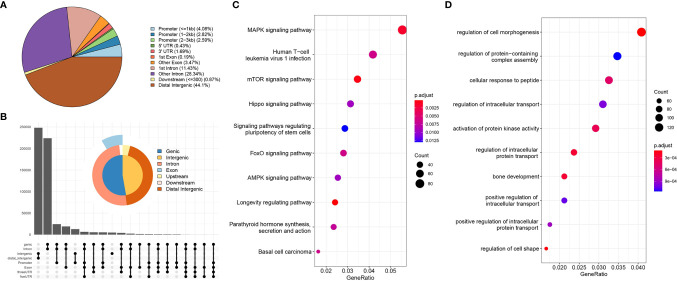
Genome characterization and enrichment analysis. [**(A)** location distribution of Peaks on the genome; **(B)** relative proportions of gene coding regions, intergenic regions, introns, exons, and upstream and downstream regions; **(C)** KEGG pathway enrichment analysis; **(D)** GO functional annotation].

Using the annotation file, we annotated the genomic coordinates corresponding to Peaks. **Figure 2A** shows the proportion of different components. As can be seen, 44% of the binding sites are in the distal intergenic region, with only 10% bound within the 3 kb region upstream and downstream of the TSS, mainly because the TSS region constitutes a small proportion of the whole genome compared to other regions. **Figure 2B** summarizes the relative enriched proportions of coding regions, intergenic regions, introns, exons and upstream and downstream regions. As shown in the figure, the downstream and distal regions have the highest proportions. **Figures 2C, D** show the GO function enrichment analysis and KEGG pathway enrichment analysis of the genes corresponding to the TSS binding sites, respectively. These show that most of the TSS binding sites are located in genes associated with regulation of cell morphology, intracellular transportation and kinase activity involved in the regulation of infection, cancer, stem cell pluripotency, the cell cycle and other related functional pathways. These functional pathways have been shown to be involved in cancer development.

### DEG Screening

**Figure 3:**
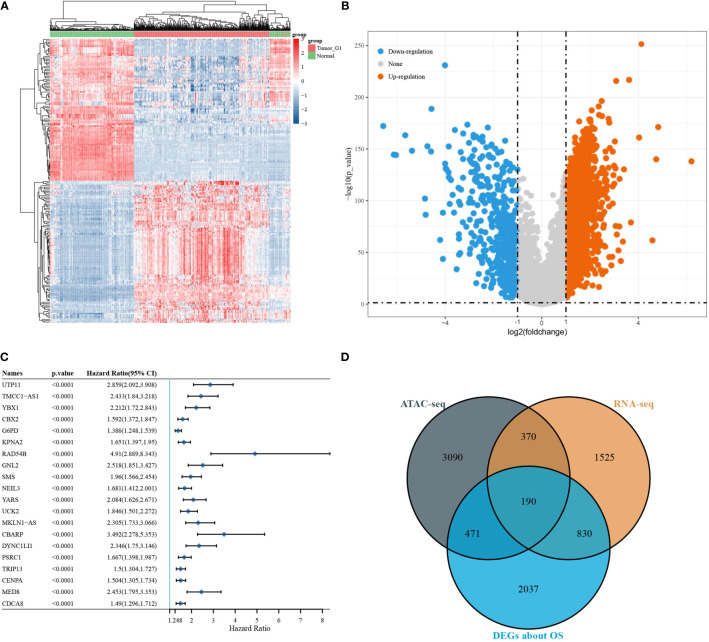
Differentially expressed gene screening. [**(A)** volcano map of differentially expressed genes; **(B)** heat map of differentially expressed genes; **(C)** DEGs associated with OS; **(D)** Venn diagram showing overlapping genes for ATAC-seq, RNA-seq, and survival-associated DEGs].

Differential expressed analysis of RNA-seq was performed on 371 LIHC tumour samples and 50 para tumour samples from TCGA database. Heat maps of the expression of each gene in each sample were drawn (**Figure 3A**). Volcano maps show the upregulated genes (*N* = 2,371) and downregulated genes (*N* = 544) obtained from the screening (**Figure 3B**). A one-way Cox analysis was used to derive 4,785 genes significantly associated with the prognosis of LIHC. The *P* values, risk factor HRs and CI column line table for the top 20 genes expressed, in addition to prognosis-related characteristics, are shown in **Figure 3C**. Finally, 190 overlapping genes were obtained by screening reproducible genes in ATAC-seq, DEGs in RNA-seq and prognosis-related genes (**Figure 3D**).

### KM Analysis of Overlapping Genes

Validation of 190 overlapping genes using the KM method yielded 126 genes that were significantly associated with OS in LIHC. Hazard coefficient HRs, with their 95% CIs and *P* values for 126 genes were derived by a log-rank test and univariate Cox proportional hazards regression (**Table 1**).

**Table 1:** 126 differential expressed genes about overall survival using Kaplan-Meier.

Genes	P	HR	Low 95%CI	High 95%CI	Genes	P	HR	Low 95%CI	High 95%CI	Genes	P	HR	Low 95%CI	High 95%CI
*NIF3L1*	0.0099	1.5796	1.1158	2.2362	*DAD1*	0.0035	1.6797	1.1856	2.3796	*EHMT2*	0.0013	1.7700	1.2485	2.5095
*SOX4*	0.0099	1.5834	1.1166	2.2452	*RPP40*	0.0035	1.6974	1.1899	2.4212	*HTATIP2*	0.0013	1.7814	1.2516	2.5355
*RSU1*	0.0097	1.5847	1.1180	2.2463	*PTGES3*	0.0034	1.6889	1.1893	2.3985	*VMA21*	0.0013	1.7713	1.2494	2.5110
*ATAD3B*	0.0096	1.5870	1.1191	2.2506	*FANCE*	0.0033	1.6949	1.1927	2.4085	*VEGFA*	0.0013	1.7829	1.2526	2.5376
*FANCI*	0.0095	1.5843	1.1191	2.2429	*UTP18*	0.0031	1.6928	1.1946	2.3988	*CDCA2*	0.0013	1.7754	1.2516	2.5184
*TSEN15*	0.0085	1.5992	1.1272	2.2688	*CDC25A*	0.0029	1.6978	1.1977	2.4068	*MELK*	0.0010	1.7944	1.2649	2.5456
*NEU1*	0.0085	1.5936	1.1262	2.2548	*VPS35*	0.0029	1.6967	1.1978	2.4034	*MGME1*	0.0010	1.8102	1.2696	2.5809
*EPS8L3*	0.0083	1.6013	1.1291	2.2712	*PYGO2*	0.0028	1.7047	1.2009	2.4199	*TOP2A*	0.0010	1.8008	1.2690	2.5555
*TOMM34*	0.0081	1.6004	1.1298	2.2670	*INCENP*	0.0028	1.6993	1.2001	2.4061	*TMEM14C*	0.0010	1.8132	1.2728	2.5829
*TBC1D16*	0.0081	1.6050	1.1310	2.2778	*IMPDH2*	0.0027	1.7082	1.2032	2.4252	*PDCL3*	0.0009	1.8052	1.2718	2.5623
*AIFM2*	0.0080	1.6041	1.1310	2.2753	*ZIC2*	0.0027	1.7128	1.2051	2.4344	*LYRM4*	0.0009	1.8287	1.2802	2.6123
*COA6*	0.0080	1.6022	1.1312	2.2694	*ECT2*	0.0027	1.7093	1.2047	2.4253	*LCMT1*	0.0009	1.8232	1.2803	2.5961
*SEMA3F*	0.0077	1.6080	1.1338	2.2806	*SLC39A10*	0.0026	1.7138	1.2063	2.4348	*GDI2*	0.0009	1.8175	1.2793	2.5820
*MRPS23*	0.0077	1.6032	1.1332	2.2681	*NAP1L1*	0.0026	1.7122	1.2062	2.4305	*HSPB11*	0.0008	1.8161	1.2807	2.5754
*ZFP64*	0.0076	1.6111	1.1349	2.2871	*PARL*	0.0024	1.7176	1.2109	2.4364	*BOD1*	0.0008	1.8279	1.2849	2.6004
*MKI67*	0.0072	1.6123	1.1377	2.2849	*CDCA4*	0.0023	1.7224	1.2146	2.4425	*CLIC1*	0.0008	1.8248	1.2848	2.5918
*LSM2*	0.0072	1.6176	1.1393	2.2966	*NOL7*	0.0022	1.7311	1.2175	2.4614	*MID1IP1*	0.0007	1.8376	1.2920	2.6135
*SLC35B2*	0.0071	1.6157	1.1396	2.2908	*JAGN1*	0.0022	1.7248	1.2161	2.4463	*HDGF*	0.0006	1.8607	1.3046	2.6538
*XPR1*	0.0070	1.6162	1.1399	2.2914	*PRCC*	0.0022	1.7291	1.2179	2.4547	*SF3B4*	0.0005	1.8608	1.3104	2.6424
*PUF60*	0.0067	1.6230	1.1435	2.3034	*MZT1*	0.0022	1.7274	1.2178	2.4503	*TXNL1*	0.0005	1.8743	1.3148	2.6720
*PRIM2*	0.0065	1.6202	1.1442	2.2941	*GTF3C6*	0.0022	1.7271	1.2178	2.4495	*HOMER3*	0.0005	1.8871	1.3226	2.6926
*LRRC1*	0.0064	1.6254	1.1460	2.3053	*GGPS1*	0.0022	1.7270	1.2178	2.4491	*RAD54L*	0.0005	1.8782	1.3203	2.6717
*CDC45*	0.0061	1.6270	1.1488	2.3043	*PSMA5*	0.0021	1.7308	1.2194	2.4568	*STMN1*	0.0004	1.8870	1.3278	2.6818
*PIGM*	0.0057	1.6411	1.1550	2.3318	*DCTPP1*	0.0021	1.7319	1.2197	2.4592	*GINS1*	0.0004	1.8888	1.3292	2.6841
*RPA2*	0.0057	1.6355	1.1541	2.3177	*RAB13*	0.0021	1.7359	1.2214	2.4671	*SLC1A5*	0.0004	1.8940	1.3309	2.6954
*BLOC1S4*	0.0057	1.6349	1.1542	2.3158	*TCF3*	0.0019	1.7393	1.2265	2.4664	*NUF2*	0.0004	1.8902	1.3308	2.6848
*POLA2*	0.0055	1.6403	1.1565	2.3265	*MARCKS*	0.0019	1.7470	1.2289	2.4835	*CD320*	0.0003	1.9044	1.3391	2.7083
*PSMD8*	0.0053	1.6473	1.1598	2.3399	*RAP2A*	0.0019	1.7425	1.2281	2.4722	*YWHAB*	0.0003	1.9211	1.3469	2.7402
*PKMYT1*	0.0052	1.6439	1.1604	2.3289	*FAF1*	0.0018	1.7390	1.2276	2.4634	*ZWINT*	0.0003	1.9159	1.3487	2.7217
*RIPK2*	0.0049	1.6611	1.1664	2.3657	*FAM189B*	0.0018	1.7568	1.2335	2.5021	*POLR3C*	0.0003	1.9588	1.3666	2.8076
*WDYHV1*	0.0045	1.6638	1.1705	2.3650	*NUDT1*	0.0018	1.7530	1.2326	2.4933	*LAPTM4B*	0.0002	1.9859	1.3835	2.8506
*DEGS1*	0.0045	1.6612	1.1705	2.3576	*ALDOA*	0.0017	1.7475	1.2322	2.4784	*EIF3M*	0.0001	1.9886	1.3966	2.8315
*ALYREF*	0.0045	1.6588	1.1702	2.3513	*RAB10*	0.0017	1.7484	1.2337	2.4776	*PARD3*	0.0001	2.0644	1.4416	2.9561
*RABIF*	0.0045	1.6622	1.1711	2.3591	*HRAS*	0.0017	1.7584	1.2368	2.5000	*EXOSC9*	0.0001	2.0730	1.4507	2.9622
*TROAP*	0.0044	1.6610	1.1710	2.3559	*RACGAP1*	0.0016	1.7556	1.2374	2.4908	*SNRPC*	0.0001	2.0650	1.4494	2.9422
*SUCO*	0.0042	1.6699	1.1757	2.3719	*KIF18A*	0.0016	1.7589	1.2390	2.4971	*CD58*	0.0001	2.0989	1.4624	3.0124
*NOL10*	0.0042	1.6663	1.1750	2.3631	*ENAH*	0.0016	1.7617	1.2399	2.5029	*GEMIN7*	0.0001	2.0853	1.4601	2.9781
*SNRPE*	0.0041	1.6725	1.1775	2.3756	*DNAJC8*	0.0015	1.7654	1.2433	2.5068	*SAC3D1*	0.0000	2.1240	1.4861	3.0357
*CNOT10*	0.0039	1.6758	1.1803	2.3795	*GCLM*	0.0015	1.7827	1.2481	2.5462	*CLTA*	0.0000	2.2159	1.5453	3.1775
*SKA1*	0.0038	1.6780	1.1820	2.3821	*PRDX6*	0.0014	1.7764	1.2486	2.5275	*ERI3*	0.0000	2.2690	1.5837	3.2509
*BUB3*	0.0037	1.6758	1.1826	2.3747	*ANO10*	0.0014	1.7668	1.2462	2.5048	*AGTRAP*	0.0000	2.2934	1.6047	3.2778
*UBAP2L*	0.0036	1.6913	1.1875	2.4086	*SLC30A6*	0.0013	1.7795	1.2510	2.5313	*UCK2*	0.0000	2.4063	1.6776	3.4514

### LASSO Model Building

**Figure 4:**
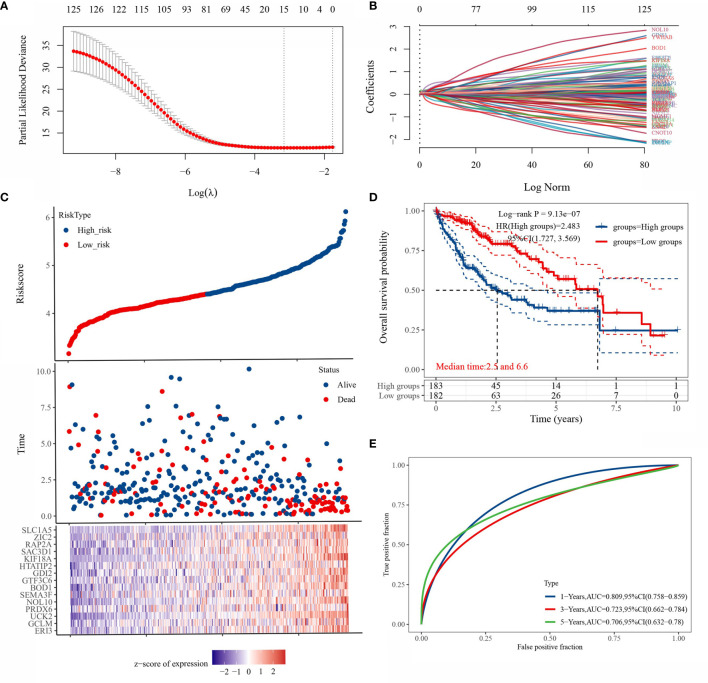
Prognostic model based on LASSO regression algorithm. [**(A)** partial likelihood deviation plotted against log(λ) using the LASSO-Cox regression model; **(B)** coefficients of selected features are shown by the lambda parameter, the horizontal axis represents the value of the independent variable lambda and the vertical axis represents the coefficients of the independent variable; [**(C)** Risk score and survival time and survival status cases; **(D)** this risk model in the TCGA KM survival curve distribution; **(E)** ROC curves with AUC at different times for this risk model].

LASSO regression was applied to 125 genes for feature selection. A prognostic model consisting of 15 gene signatures was obtained after 10-fold cross-validation (**Figures 4A, B**). The complete gene names of 15 genes are shown in **Supplementary Table 1**. **Figure 4C** shows the association between the risk score and survival time with survival status in the TCGA dataset. **Figure 4D** shows the distribution of KM curves of the risk model in the TCGA dataset. The gene signature model was divided into high-risk and low-risk groups according to the risk score, with a HR of 2.483 representing a risk factor. **Figure 4E** shows the ROC curves and AUC of the risk model at different times. The AUC values at 1, 3 and 5 years were 0.809, 0.723 and 0.706, respectively, indicating that the model has a strong predictive ability.

### Expression of 15 Gene Signatures in LIHC

**Figure 5:**
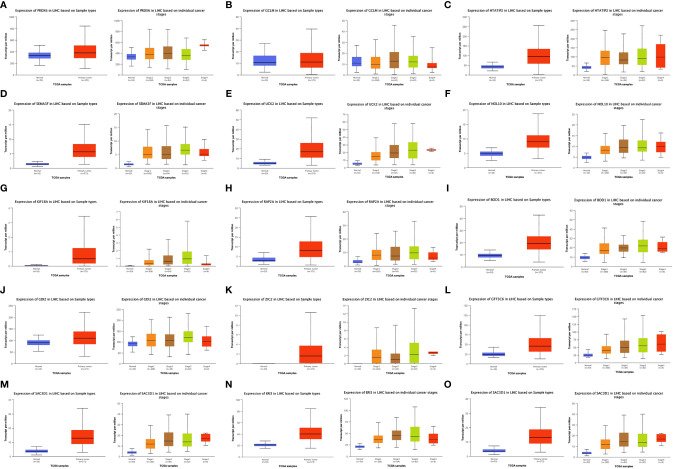
Expression of 15 Signature genes in LIHC in cancer/normal and different stages. [**(A)** PRDX6; **(B)** GCLM; **(C)** HTATIP2; **(D)** SEMA3F; **(E)** UCK2; **(F)** NOL10; **(G)** KIF18A; **(H)** RAP2A; **(I)** BOD1; **(J)** GDI2; **(K)** ZIC2; **(L)** GTF3C6; **(M)** SLC1A5; **(N)** ERI3; **(O)** SAC3D1].

**Figures 5A–O** show the expression of the 15 gene signatures [PRDX6 (**Figure 5A**), GCLM (**Figure 5B**), HTATIP2 (**Figure 5C**), SEMA3F (**Figure 5D**), UCK2 (**Figure 5E**), NOL10 (**Figure 5F**), KIF18A (**Figure 5G**), RAP2A (**Figure 5H**), BOD1 (**Figure 5I**), GDI2 (**Figure 5J**), ZIC2 (**Figure 5K**), GTF3C6 (**Figure 5L**), SLC1A5 (**Figure 5M**), ERI3 (**Figure 5N**) and SAC3D1 (**Figure 5O**)] in LIHC cancer tissues relative to that in paraneoplastic tissues and different cancer stages. **Table 2** shows the statistical significance (P value) of gene expression in different tissues and different stages. Fifteen genes were upregulated in the LIHC tissues. Most of these genes were not significantly expressed in different LIHC stages.

**Table 2:** Statistical significance of 15 genes.

	*PRDX6*	*GCLM*	*HTATIP2*	*SEMA3F*	*UCK2*	*NOL10*	*KIF18A*	*RAP2A*	*BOD1*	*GDI2*	*ZIC2*	*GTF3C6*	*SLC1A5*	*ERI3*	*SAC3D1*
Normal *vs* Primary	3.53E-10	2.22E-04	1.62E-12	<1E-12	1.62E-12	<1E-12	1.62E-12	<1E-12	1.62E-12	1.65E-12	<1E-12	1.62E-12	5.78E-10	1.62E-12	1.62E-12
Normal *vs* Stage1	3.76E-07	1.59E-01	<1E-12	<1E-12	<1E-12	1.62E-12	1.62E-12	1.62E-12	<1E-12	5.30E-07	1.62E-12	<1E-12	1.99E-04	<1E-12	<1E-12
Normal *vs* Stage2	1.00E-04	9.44E-03	1.87E-10	1.62E-12	1.62E-12	1.62E-12	1.33E-07	4.26E-10	1.62E-12	2.19E-05	4.32E-09	1.45E-13	5.74E-03	1.62E-12	1.62E-12
Normal *vs* Stage3	6.64E-03	5.04E-03	1.85E-12	<1E-12	<1E-12	1.63E-12	6.44E-10	9.48E-11	<1E-12	1.41E-10	2.34E-11	1.91E-12	8.03E-05	1.64E-12	1.62E-12
Normal *vs* Stage4	9.72E-07	5.29E-01	1.07E-01	1.00E-02	1.14E-03	3.01E-02	1.38E-01	8.15E-02	8.76E-03	3.42E-01	5.50E-02	3.82E-02	6.53E-02	3.51E-02	9.62E-04
Stage1 *vs* Stage2	4.70E-01	4.81E-02	4.52E-01	8.30E-01	1.48E-02	3.12E-03	2.83E-02	4.52E-01	7.39E-02	1.41E-01	1.39E-01	1.62E-02	1.03E-01	5.63E-04	2.90E-03
Stage1 *vs* Stage3	2.52E-01	3.13E-02	9.41E-01	4.28E-02	3.80E-04	3.45E-04	1.76E-04	4.33E-02	3.46E-03	3.17E-04	7.20E-03	6.56E-04	1.23E-02	2.00E-03	1.54E-03
Stage1 *vs* Stage4	3.63E-01	6.71E-01	7.88E-01	6.91E-01	4.41E-01	4.67E-01	9.19E-01	9.83E-01	7.63E-01	8.82E-01	6.09E-01	1.56E-01	6.12E-01	9.15E-01	2.29E-01
Stage2 *vs* Stage3	1.21E-01	9.63E-01	4.58E-01	3.87E-02	3.32E-01	1.36E-01	1.16E-01	2.18E-01	1.79E-01	1.10E-01	3.03E-01	2.09E-01	7.58E-01	4.29E-01	5.86E-01
Stage2 *vs* Stage4	6.06E-01	8.47E-01	9.79E-01	7.13E-01	9.60E-01	8.13E-01	5.70E-01	8.38E-01	7.78E-01	5.97E-01	9.45E-01	6.91E-01	4.70E-01	2.78E-01	9.78E-01
Stage3 *vs* Stage4	1.76E-01	8.22E-01	7.72E-01	2.74E-01	6.85E-01	5.26E-01	2.98E-01	5.53E-01	5.07E-01	2.28E-01	6.54E-01	9.21E-01	2.50E-01	3.38E-01	8.68E-01

### Prognostic Analysis of 15 Gene Signatures in LIHC Patients

**Figure 6:**
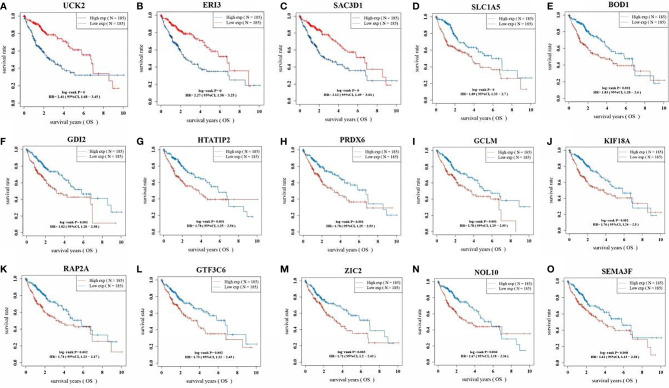
Kaplan Meier curve of 15 gene signatures in LIHC patients. (**A**: *UCK2*; **B**: *ERI3*; **C**: *SAC3D1*; **D**: *SLC1A5*; **E**: *BOD1*; **F**: *GDI2*; **G**: *HTATIP2*; **H**: *PRDX6*; **I**: *GCLM*; **J**: *KIF18A*; **K**: *RAP2A*; **L**: *GTF3C6*; **M**: *ZIC2*; **N**: *NOL10*; **O**: *SEMA3F*).

The KM analysis of the survival prognosis of the 15 gene signatures in LIHC patients showed that high expression of all 15 gene signatures was significantly associated with a poor prognosis in LIHC patients (**Figure 6**).

### Correlation of 15 Gene Signatures With Immune Cell Infiltration

**Figure 7:**
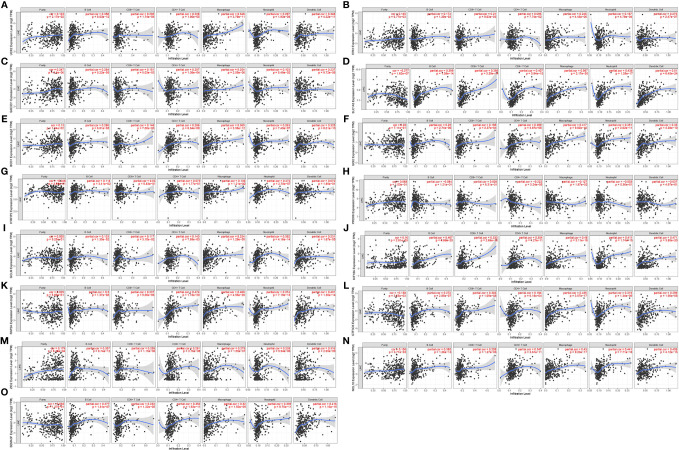
Correlation of 15 gene signatures with immune cell infiltration [**(A)** UCK2 correlates with immune cell infiltration; **(B)** ERI3 correlates with immune cell infiltration; **(C)** SAC3D1 correlates with immune cell infiltration; **(D)** SLC1A5 correlates with immune cell infiltration; **(E)** BOD1 correlates with immune cell infiltration; **(F)** GDI2 correlates with immune cell infiltration; **(G)** HTATIP2 correlates with immune cell infiltration; **(H)** PRDX6 correlated with immune cell infiltration; **(I)** GCLM correlated with immune cell infiltration; **(J)** KIF18A correlated with immune cell infiltration; **(K)** RAP2A correlated with immune cell infiltration; **(L)** GTF3C6 correlated with immune cell infiltration; **(M)** ZIC2 correlated with immune cell infiltration; **(N)** NOL10 correlated with immune cell infiltration; **(O)**. SEMA3F correlated with immune cell infiltration).

**Figures 7A–O** shows the correlation of the 15 gene signatures with immune cell infiltration levels in LIHC. All the gene signatures other than those of *PRDX6* and *HTATIP2* were correlated with tumour purity and B cell, CD4+ T cell, CD8+ T cell, macrophage, neutrophil and dendritic cell levels. Infiltration levels were all significantly and positively correlated.

### Validation of the Protein Expression of 15 Gene Signatures

**Figure 8:**
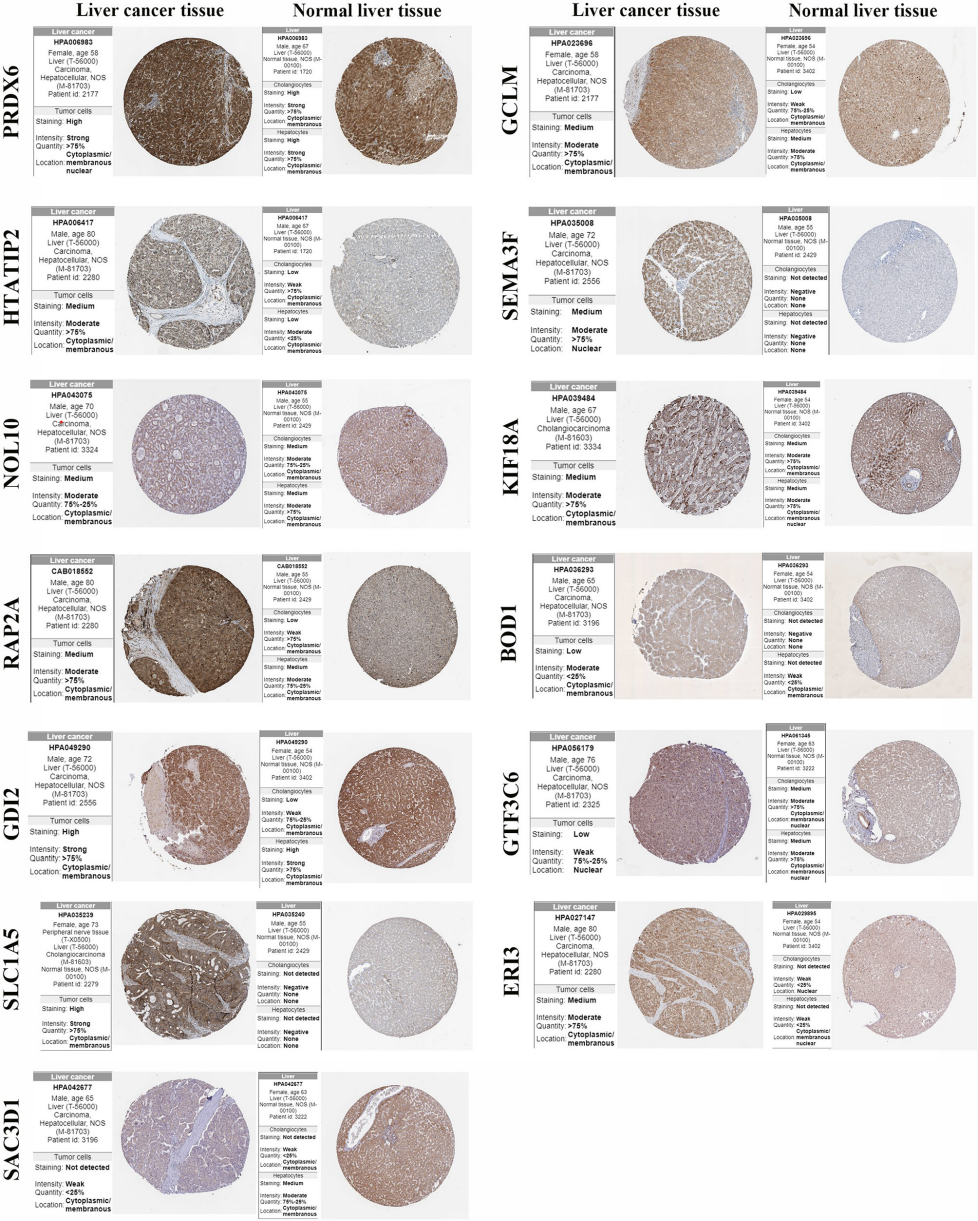
Protein expression of gene signature in hepatocellular carcinoma tissue and normal liver tissue.

The protein expression of the 15 gene signatures in hepatocellular carcinoma tissues and normal liver tissues was verified using the HPA online database (**Figure 8**). The results showed that *PRDX6*, *GCLM*, *HTATIP2*, *NOL10*, *KIF18A*, *RAP2A* and *GDI2* were highly expressed in the hepatocellular liver cancer tissues compared to the normal liver tissues. *SEMA3F*, *BOD1*, *SLC1A5* and *ERI3* were not detected in cholangiocytes and hepatocytes. *SAC3D1* was not detected in hepatocellular liver cancer tissues. In addition, *UCK2* and *ZIC2* were not detected in the hepatocellular carcinoma samples in the protein expression data.

### GO and KEGG Pathway Enrichment Analysis of 15 Gene Signatures

**Figure 9:**
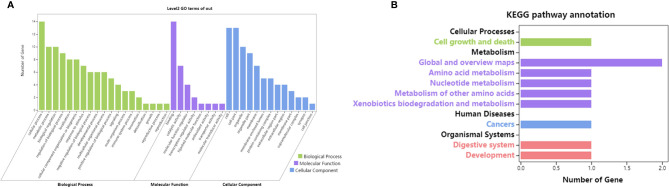
GO and KEGG enrichment of 15 gene signatures. [**(A)** GO enrichment of 15 gene signatures; **(B)** KEGG enrichment of 15 gene signatures].

In the GO and KEGG enrichment analyses of the 15 gene signatures, cellular processes, binding, metabolism and cancer were enriched (**Figure 9**).

## Discussion

Tumours usually have altered epigenetic patterns, and epigenetic regulators are frequently mutated in cancer (16). ATAC-seq is an innovative technique for epigenetic studies that cleaves nuclear chromatin regions that are open at a particular condition location by transposes and thus obtains the regulatory sequences of all active transcripts in the genome at that particular condition (12). In the present study, we performed genomic interval distribution statistics using ATAC-seq for peaks identified in different LIHC samples, while comparing chromatin open site differences between different samples. Furthermore, we conducted GO and KEGG enrichment analysis for genes adjacent to these differential sites. A portion of genes associated with the GO functional enrichment pathway was identified. Subsequently, RNA-seq was used to analyse DEGs in the LIHC samples, and a one-way Cox analysis was performed to analyse genes significantly associated with the prognosis in LIHC. In total, 190 key genes were obtained by overlapping the genes screened using the three methods. Further validation by KM analysis yielded 125 key genes. However, due to the large number of 125 key genes, featured genes needed to be further extracted.

To take account of the dimensional catastrophe problem, we used LASSO regression analysis for further analysis of the gene set. By using a penalty function to compress the regression coefficients of the independent variables, LASSO provides good shrinkage control and can compress the regression coefficients of some independent variables to 0 (17). Finally, we obtained a sparse model and then obtained genes with a higher significant correlation with the survival prognosis of liver cancer patients. According to 10-fold cross-validation, a penalty parameter, λ = 0.0425, was finally selected, and then λ was substituted into the regression equation of LASSO to ensure that the sum of the absolute values of the regression coefficients of all independent variables was less than or equal to the selected penalty parameter λ. Finally, the regression coefficients of a large number of genetic variables were compressed to 0, and genes with regression coefficients except that were selected and subjected to LASSO regression.

Fifteen gene signatures were identified: *PRDX6*, *GCLM*, *HTATIP2*, *SEMA3F*, *UCK2*, *NOL10*, *KIF18A*, *RAP2A*, *BOD1*, *GDI2*, *ZIC2*, *GTF3C6*, *SLC1A5*, *ERI3* and *SAC3D1*. We generated a prognostic index for each sample for the risk scores of each gene in each sample and then divided the samples into high risk and low risk according to the prognostic index to analyse the overall survival time of the samples. The results revealed that the survival of patients classified as high risk was significantly worse than that of patients classified as low risk. The prognostic model had good predictive power.

Subsequently, we investigated the expression of each gene in LIHC patients and patients with various LIHC disease stages and found that each gene was significantly upregulated in cancerous tissues in LIHC patients compared with paracancerous tissues and that highly expressed genes were significantly associated with a poor patient prognosis. This finding suggested that these genes can be used as prognostic predictive biomarkers for LIHC and that the prediction model combining these genes performs well.

We also analysed the correlation between gene expression and immune cell infiltration levels using TIMER data and found that other than *PRDX6* and *HTATIP2*, all other 13 gene signatures were significantly and positively correlated with tumour purity and B cell, CD4+ T cell, CD8+ T cell, macrophage, neutrophil and dendritic cell infiltration levels. This finding suggested that these gene signatures may influence tumour progression by regulating the tumour microenvironment. *PRDX6* is a unique bifunctional enzyme, which reduces both water-soluble and lipid-soluble peroxides and has unique phospholipase A2 activity (18). A previous study reported that inhibition of *PRDX6* expression promoted apoptosis in Hepa1-6 cells (19). Another study reported that interventional treatment of primary liver cancer can reduce serum HTATIP2/TIP30 and B7-H4 levels, improve liver function and quality of life and prolong the survival times of patients (20). A number of studies reported that *SEMA3F*, *UCK2*, *NOL10*, *RAP2A*, *ZIC2* and *SAC3D1* are associated with prognosis and tumour immune infiltration in hepatocellular carcinomas (21–25). In addition, KIF18A was reported to be associated with tumour immune cell infiltration in adrenocortical carcinomas (26). *SLC1A5* may serve as a potential target for enhancing anti-tumour immunity in the tumour microenvironment (27). According to the literature, *GCLM* gene polymorphisms are associated with hepatitis B virus-induced liver disease (28). There is limited research on *BOD1, GDI2, GTF3C6* and *ERI3’* impact on hepatocellular carcinomas. *BOD1* is a novel mitogenic protein required for chromosomal localization (29). It may act as a unique cytoplasmic interacting protein to regulate signal pathway,thereby having potential in the treatment of various diseases, including cancer (30). *GDI2* belongs to a small family of chaperone proteins expressed mainly in hematopoietic, endothelial and epithelial cells (31). *GDI2* expression is abnormal in many cancer types, including pancreatic, ovarian, gastric and oesophageal squamous cell carcinomas (32–34). There are few studies on *GTF3C6* and *ERI3.* One previous study found that an integrated model based on a six-gene signature (35) or a novel ferroptosis-related gene signature (36) could predict OS in patients with hepatocellular carcinomas. Most previous studies mainly used only RNA-seq to perform bioinformatic analyses. In contrast, we used ATAC-seq and RNA-seq, which are mutually authenticating, further strengthens the findings of the present study.

In summary, we obtained 15 gene signatures associated with the survival prognosis of hepatocellular carcinoma patients based on ATAC-seq and RNA-seq integration analysis and LASSO regression analysis. Due to the limitations of the experimental conditions, it was not possible to obtain a detailed understanding of the specific mechanism of the action of each of the 15 hepatocellular carcinoma signature genes. Thus far, there have been few studies on *BOD1*, *GDI2*, *GTF3C6* and *ERI3*’ impact on hepatocellular carcinomas. The findings of our study provide theoretical basis and directions for future studies. The 15 gene signatures of a survival prognosis in hepatocellular carcinoma patients identified herein may contribute to the development of targeted treatment for hepatocellular carcinoma patients.

## Data Availability Statement

The original contributions presented in the study are included in the article/**Supplementary Material**. Further inquiries can be directed to the corresponding author.

## Author Contributions

We contributed equally for this work. All authors contributed to the article and approved the submitted version.

## Conflict of Interest

The authors declare that the research was conducted in the absence of any commercial or financial relationships that could be construed as a potential conflict of interest.

## Publisher’s Note

All claims expressed in this article are solely those of the authors and do not necessarily represent those of their affiliated organizations, or those of the publisher, the editors and the reviewers. Any product that may be evaluated in this article, or claim that may be made by its manufacturer, is not guaranteed or endorsed by the publisher.
