# Supplementary material for: Bioinformatics Analysis Using ATAC-seq and RNA-seq for the Identification of 15 Gene Signatures Associated With the Prediction of Prognosis in Hepatocellular Carcinoma
Source: Front Oncol. 2021 Oct 25;11:726551. doi: 10.3389/fonc.2021.726551 (PMC8573251; doi:10.3389/fonc.2021.726551)
Supplement: Supplementary file 1 [file Table_1.docx]

| **Supplementary Table 1.Complete gene name of 15 genes** | |
| --- | --- |
| Gene symbol | Complete gene name |
| *PRDX6* | Acidic Calcium-Independent Phospholipase A2 |
| *GCLM* | Gamma-Glutamylcysteine Synthetase Regulatory Subunit |
| *HTATIP2* | HIV-1 Tat Interactive Protein 2 |
| *SEMA3F* | Semaphorin-3F |
| *UCK2* | Uridine Monophosphokinase 2 |
| *NOL10* | Polyglutamine Binding Protein 5 |
| *KIF18A* | Kinesin-Like Protein KIF18A |
| *RAP2A* | Ras-Related Protein Rap-2a |
| *BOD1* | Biorientation Of Chromosomes In Cell Division Protein 1 |
| *GDI2* | Guanosine Diphosphate Dissociation Inhibitor 2 |
| *ZIC2* | Zinc Finger Protein Of The Cerebellum 2 |
| *GTF3C6* | General Transcription Factor IIIC, Polypeptide 6 |
| *SLC1A5* | Solute Carrier Family 1 (Neutral Amino Acid Transporter), Member 5 |
| *ERI3* | ERI1 Exoribonuclease 3 |
| *SAC3D1* | SAC3 Homology Domain-Containing Protein 1 |
